# The mechanism of neuroprotective effect of *Viola odorata* against serum/glucose deprivation-induced PC12 cell death

**DOI:** 10.22038/AJP.2019.13098

**Published:** 2019

**Authors:** Zahra Tayarani-Najaran, Rezvan Yazdian-Robati, Elaheh Amini, Farzaneh Salek, Fatemeh Arasteh, Seyed Ahmad Emami

**Affiliations:** 1 *Biotechnology Research Center, Pharmaceutical Technology Institute, Mashhad University of Medical Sciences, Mashhad, Iran*; 2 *Molecular and Cell Biology Research Center, Faculty of Medicine, Mazandaran University of Medical Sciences, Sari, Iran*; 3 *Department of Cellular and Molecular Biology, Faculty of Biological Sciences, Kharazmi University, Tehran, Iran*; 4 *Department of Biology, Faculty of Sciences, Mashhad Branch, Islamic Azad University, Mashhad, Iran*; 5 *Department of Pharmaceutical Biotechnology, School of Pharmacy, Mashhad University of Medical Sciences, Mashhad, Iran*; 6 *Department of Traditional Pharmacy, School of Pharmacy, Mashhad University of Medical Sciences, Mashhad, Iran*

**Keywords:** Viola odorata, Violaceae, PC12 cells, ROS, SGD

## Abstract

**Objective:**

Oxidative stress is associated with the pathogenesis of brain ischemia and other neurodegenerative disorders. Previous researches have shown the antioxidant activity of *Viola* *odorata* L. In this project, we studied neuro-protective and reactive oxygen species (ROS) scavenging activities of methanol (MeOH) extract and other fractions isolated from *V. odorata *in PC12 cell line in serum/glucose deprivation (SGD) condition.

**Materials and Methods:**

The PC12 neuronal cells were pretreated for 6 hr with MeOH extract and fractions of *V. odorata *(1 to 25 μg/ml) followed by 24 hr incubation under SGD condition. Cell viability was measured by Alamar Blue^® ^assay. The level of ROS was calculated using DCFH-DA. Also, Bax/Bcl-2 protein ratio was analyzed by western blot assay.

**Results:**

SGD condition significantly decreased cells viability (p<0.001). Pretreatment with EtOAc (12.5 and 25 µg/ml), BuOH (12, 25, 50 µg/ml) and CH_2_Cl_2 _(1.5 µg/ml) fractions of *V. odorata* reduced SGD-induced cytotoxicity. MeOH extract could not increase the viability significantly. All four semi polar fractions (EtOAc, BuOH, CH2Cl2 and MeOH) decreased SGD-induced ROS production and changed Bax/Bcl-2 ratio.

**Conclusion:**

*V. odorata* showed promising effects against SGD condition; further mechanistic and clinical studies are warranted before application of *V. odorata* as a neuro-protective agent.

## Introduction

Blocking blood flow to the brain, even for a short period, can cause poor supply of glucose, oxygen and nutrients resulting in loss of energy supply and focal cerebral ischemia (Sims and Muyderman, 2010). Multiple factors have been proposed to participate in ischemia-induced neuronal cell damages such as apoptosis induction, kinase activation and production of reactive oxygen species (ROS) (Ekshyyan and Aw, 2004). ROS are generated from the mitochondrial electron transport chain when cells utilize oxygen to produce energy (Collins et al., 1998). High level of ROS is strongly linked with pathogenesis of ischemia-induced neuronal cell damage and also causes other neurodegenerative disorders (Mórocz et al., 2002). The human body employs different mechanisms to respond oxidative stress by producing antioxidants. These antioxidants as free radical scavengers, prevent the damages caused by ROS (Pham-Huy et al., 2008). Recently, several studies focused on finding antioxidant phytochemicals (Bors et al., 1984; Mousavi et al., 2010; Hosseinzadeh et al., 2009). Serum/glucose deprivation (SGD) as an excellent *in vitro* model was exploited for understanding the molecular mechanisms of neuronal injury during brain ischemia as well as studying neuroprotective drugs against ischemia-induced brain damage (Woronowicz et al., 2007). PC12 cells derived from a pheochromocytoma tumor of the rat adrenal medulla, has been used as a suitable model for *in vitro* studying of the mechanisms involved in neuronal cell death (Hillion et al., 2005). *Viola odorata* L. from the Violaceae family, is found all over the world. The plant is native to Asia, North Africa and Europe and is usually known as sweet violet (English name) or “*banafsheh*” (Persian name) (Javadi et al., 2015). *V. odorata* is traditionally used as a medicinal plant in the Islamic traditional medicine for treating childhood eczema, mouth infections, anxiety, insomnia, high blood pressure; also, pharmacological studies have reported that this plant has anti-inflammatory, antibacterial and antioxidant activities (Tayarani-Najaran et al., 2014). A previous study showed that hydroalcoholic extract of *V. odorata* possess an antioxidant property and it can protect neuronal cells against SGD-induced cell death but the molecular mechanism of this protective effect was not discussed (Mousavi et al., 2010).

In this study, we evaluated methanol (MeOH) extract of *V. odorata *as well as its different fractions including dichloromethane (CH_2_Cl_2_), ethyl acetate (EtOAc), *n*-butanol (BuOH) and water (H_2_O) for potential neuro-protective effect against SGD-induced PC12 cell death. Moreover, ROS scavenging properties of the MeOH extract and fractions were studied. The effect of the extract on Bax/Bcl-2 level was also studied.

## Materials and Methods

**Plant materials**

*V. odorata* was collected in July 2013, from Mashhad, Khorasan Razavi province of Iran and identified and stored by Mrs. M. Souzani in the Herbarium of School of Pharmacy, Mashhad University of Medical Sciences with a voucher specimen (No.12855). Plant materials were dried in shadow at room temperature and coarsely ground into a fine powder before extraction. Using a percolation method, 327 g of powdered leaves were incubated in 95% MeOH at controlled room temperature for 24 hr. In a decantation funnel, the percolated mixture was extracted and then, concentrated in a rotary vacuum. Using a freeze dry process, the solvent was completely removed and 50 g of crude solid extract was obtained (yield 15.3%). Fractionation of the MeOH extract was further performed using *n-*hexane, CH_2_Cl_2_, EtOAc, BuOH and H_2_O.

**Cell line and reagents**

PC12 cell line was purchased from Pasteur Institute (Tehran, Iran). AlamarBlue® (resazurin) was bought from Sigma (Saint Louis, MO, USA); Bcl-2 rabbit mAb (Cell Signaling, #2870) and Bax antibody (Cell Signaling, #2772) were bought from Cell Signaling technology (Boston, USA); ECL western blotting detection reagent was purchased from Bio-Rad (USA); High glucose Dulbecco’s modified Eagle’s Medium (DMEM, 4.5 g/L), glucose-free DMEM, penicillin-streptomycin, and fetal bovine serum (FBS) were bought from GIBCO (Grand Island, NY, USA).

**Induction of cell death by serum/glucose deprivation**

At first, PC12 cells were treated with MeOH extract and fractions *V. odorata* (0 to 25 µg/ml) for 6 hr. Then, cells exposed to SGD, were switched from the standard culture (high glucose DMEM, 4.5 g/L) to the glucose-free DMEM (0 g/L) supplemented with 100 U/mL penicillin and 100 U/mL streptomycin (Mousavi et al., 2010) overnight.

**Cell viability**

Cell viability was measured by Alamar Blue^® ^method using resazurin. Resazurin is a water-soluble indicator of oxidation-reduction which is also known as diazo-resorcinol, azoresorcin, resazoin, and resazurine (Anoopkumar-Dukie et al., 2005). Resazurin is non-toxic, and stable in culture medium and can permeate through the cell membrane. In viable cells, resazurin converts to resorufin, and produces a florescent purple color but a blue nonflorescent color in dead cells (Tayarani-Najaran et al., 2013) 

At the end of incubation under SGD condition, the Alamar Blue^® ^was added to the cell media at a final concentration of 0.5 mg/ml. The cells were incubated in a humidified atmosphere containing 5% CO_2_ at 37°C. After 4 hr, the absorbance was measured at 570 and 600 nm in a Synergy H4 microplate reader (BioTek, USA). The IC_50_ values were analyzed (Graph Pad prism 5 software) and the viability of cells in three independent experiments is presented as mean±SD.

**Measurement of** **intracellular reactive oxygen species (ROS)**

Intracellular ROS were evaluated by quantitating the fluorescent signal of 2′, 7′-dichlorofluorescein diacetate (DCFH-DA). Upon oxidation by ROS, DCFH-DA is deacetylated by nonspecific esterases, generating non-fluorescent 2′, 7′-dichlorofluorescin (DCFH), which is oxidized to a fluorescent compound, DCF (Galato et al., 2001). In order to determine the level of intracellular ROS, PC12 cells were seeded into 96-well culture plate (10^5 ^cells/well) and pretreated with MeOH extract and fractions of *V. odorata* for 4 hr. After 4 hr in SGD condition, cells were incubated with 50 µl H_2_O_2 _(24 mM) at 37°C for 30 min. Then, 50 µl of DCFH-DA was mixed with the cells and the fluorescence intensity of DCF was measured at 528 nm emission and 485 nm excitation using a Synergy H4 microplate reader (BioTek, USA). 

**Western blot analysis**

PC12 cells treated with EC50 optimum concentration of the MeOH extract and the other fractions of *V. odorata,* were lysed using cell lysis buffer (Tris-HCl 50 µM, NaCl 150 mM, NP-40 1%, EDTA 1 mM, SDS 0.2%, protease inhibitor 1%, phosphatase inhibitor 1% and phenylmethylsulfonyl fluoride (PMSF) 1 mM, ice-incubation for 30 min) and pelleted by centrifugation (12000 rpm, 10 min, 4°C). After estimation of protein levels by Bovine Serum Albumin (BSA), equal amounts of protein extracts (50 µg) from cells treated with MeOH extract and fractions of *V. odorata*, were loaded on 10% SDS gel and blotted on polyvinylidene fluoride (PVDF) membrane by a wet transfer system (250 mA for 2 hr). Blots were blocked with bovine serum albumin and the membrane was incubated overnight at 4°C with specific anti-protein primary antibodies. The membrane was then incubated with an anti-primary secondary antibody conjugated with horseradish peroxidase for 2 hr at 37°C. Protein bands were detected by means of enhanced chemiluminescence (Pierce ECL western blotting substrate) and Alliance gel doc (Alliance Gel doc, UK).

**Statistical analysis**

One way analysis of variance (ANOVA) and Dunnett’s *post hoc* test were performed for data analysis. All results are expressed as mean±SEM and p values below 0.05 were considered statistically significant.

## Results

**Effects of different** ***V. odorata*** **extracts on cell viability**

The viability of PC12 cells after treatment with MeOH extract and fractions of *V. odorata *for 6 hr, was measured by Alamar Blue^® ^assay. 

**Figure 1:**
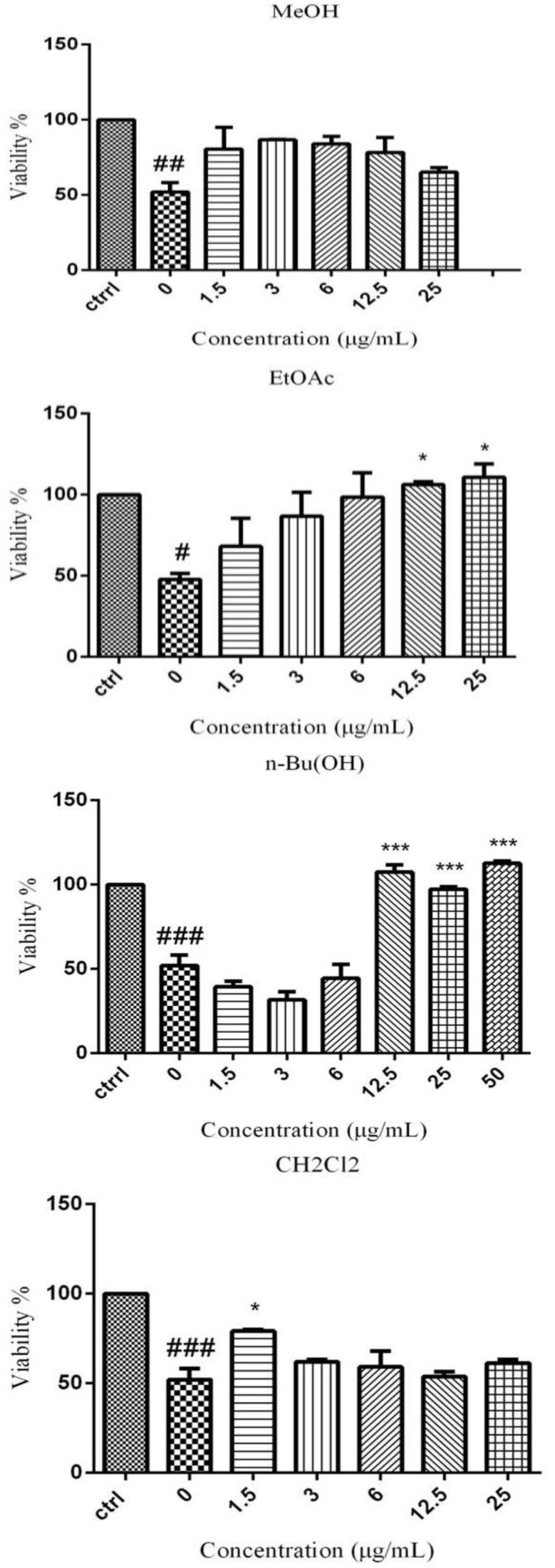
Effect of MeOH extract and fractions of *V. odorata *on viability of PC12 cells under SGD condition. The cells were pretreated for 6 hr with MeOH extract and other fractions of *V. odorata *and then exposed to SGD for an additional 24 hr. The cell viability is expressed as the percentage of cells cultured in high-glucose medium (control). The data presented are means±SEM of three independent experiments (n=3). #p<0.05, ##p<0.01 and ###p<0.001 serum/glucose deprivation (SGD) compared to control. *p<0.05, and ***p<0.001 compared to concentration of 0 μg/ml in SGD condition

After 6 hr incubation, SGD condition significantly decreased cell viability as compared to control condition. As shown in Figure 1, pretreatment with EtOAc (12.5 and 25 µg/ml), BuOH (12, 25, 50 µg/ml) and CH_2_Cl_2 _(1.5 µg/ml) could significantly increase the cell viability. MeOH extract could not increase the viability significantly. 

**Effects of MeOH extract and different fractions of** ***V. odorata*** **on ROS production**

**Figure 2:**
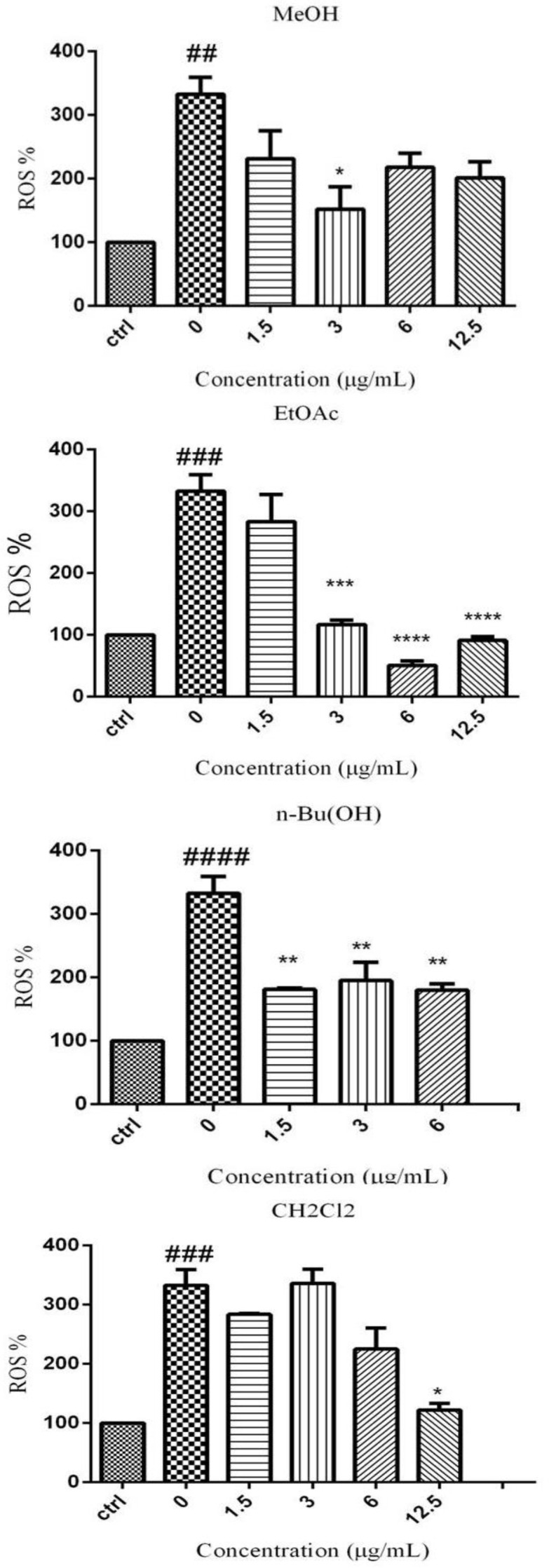
Effect of MeOH extract and different fractions of* V. odorata *on the level of ROS in PC12 cells under SGD condition. The cells were pretreated with MeOH extract and fractions of* V. odorata *for 6 hr and then exposed to SGD for an additional 24 hr. The values represent 5 independent experiments. ##p<0.01, ### p<0.001, ####p<0.0001 serum/glucose deprivation (SGD) compared to control; *p<0.05, **p<0.01, and ***p<0.001 compared to concentration of 0 μg/ml in SGD condition

Generation of intracellular ROS in PC12 cells was significantly enhanced after 4 hr of SGD insult in all groups, as compared to control. As shown in Figure 2, pre-incubation with MeOH (3 µg/ml), EtOAc (3, 6, and 12.5 μg/ml), BuOH (1.5, 3, and 6 μg/ml) and CH_2_Cl_2 _(12.5 µg/ml) extracts significantly decreased SGD-induced ROS production.

**Figure 3:**
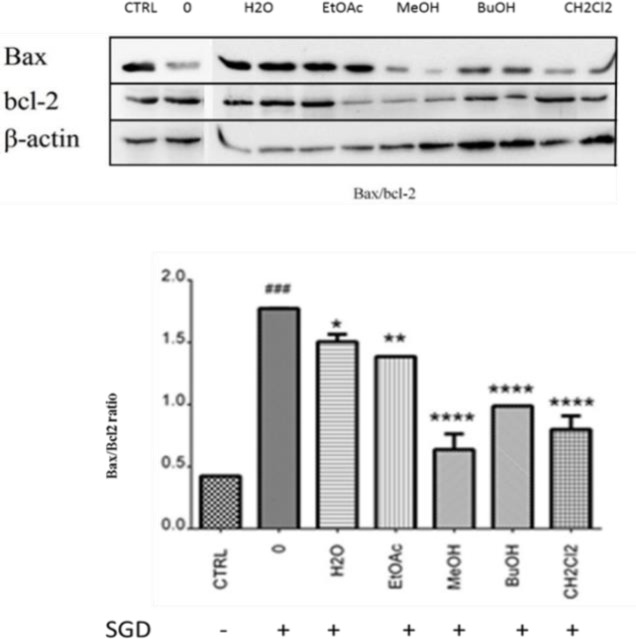
Western blot analysis of Bax/Bcl-2 ratio in cell treated with MeOH extract and other fractions of* V. odorata*. (0=SGD condition without any treatment) β-Actin was used as a loading control. All western blots were representative of 3 independent experiments

**Effects of different** ***V. odorata*** **extracts on Bax/Bcl-2 ratio**

Results of western blot analysis of Bax/Bcl-2 ratio in cell treated with MeOH extract and other fractions of *V. odorata* are shown in Figure 3. Our results indicated that Bax/Bcl-2 ratio after treatment with MeOH extract and other fractions of *V. odorata*, reduced significantly in SGD-induced PC12 cells. The MeOH extract had the most significant effect in terms of protection against apoptotic insults.

## Discussion

In acute ischemia, high level of ROS causes oxidative damage to especially polyunsaturated fatty acids of lipid membranes and other cellular macromolecules (Piantadosi and Zhang, 1996). Antioxidants can scavenge free radicals and reduce the alterations of cell membrane permeability caused by oxidative damage, so they are beneficial in prevention of problems associated with brain ischemia (Halliwell, 1992; Pham-Huy et al., 2008). Previous studies have shown some protective effects of *V. odorata* on mammalian cells (Qadir et al., 2014; Koochek et al., 2003). The water extract as well as phenolic extract of *V. odorata *was also reported to neutralize DPPH radicals in a dose-dependent manner (Stojković et al., 2011; Ebrahimzadeh et al., 2010). In the present work, our results revealed that MeOH extract and semipolar fractions of* V. odorata *are able to prevent neuronal damage induced by SGD. SGD condition decreased the cell viability for about 50%, which was comparable to previous reports (Alinejad et al., 2013; Sadeghnia et al., 2012). In addition, our results showed that EtOAc (12.5 and 25 µg/ml), BuOH (12, 25, and 50 µg/ml) and CH_2_Cl_2_ (1.5 µg/ml) could increase the cell viability significantly (Figure 1). While the SGD condition increased the intracellular ROS level but pretreatment with MeOH extract and fractions of* V. odorata* could block the SGD-induced ROS production and this may be due to neuroprotective effect of *V. odorata* (Figure 2). Also, in another study, it was reported that MeOH extract of *V. odorata* can scavenge free radicals and has an antioxidant property (Ebrahimzadeh et al., 2010)

Besides oxidative stress, different pathological events are activated such as transcription of large quantities of mRNAs causing alterations in the expression of numerous genes involved in cell death signaling either necrosis or apoptosis (Ebrahimzadeh et al., 2010; Alinejad et al., 2013). Apoptosis is programmed cell death that ensures cell homeostasis. Different proteins are involved in this key physiological process (Wyllie, 1972). Bcl-2 protein is one of the proteins in apoptosis pathway that can protect cell against external insults and inhibit or delay apoptosis induced by various stimuli such as free radicals (Reed et al., 1996; Sato et al., 1994). In contrast, Bax protein generates death signals and antagonizes Bc1-2 anti-apoptotic function of Bcl-2. The ratio of anti-apoptotic Bcl-2 to pro-apoptotic Bax in a cell determines whether apoptosis occurs or not (Hockenbery et al., 1993). In our study, following pretreatment of PC12 cells with MeOH extract and other fractions of* V. odorata*, Bax/Bcl-2 ratio was decreased significantly in SGD cells representing decreased expression of pro-apoptotic Bax or increased expression of anti-apoptotic Bcl-2. The MeOH extract showed the most significant effect in terms of protection against apoptotic insults.

Comparing the protective effect of MeOH extract and fractions of* V. odorata*, it is important to note that fractions which are fractionated using semi polar solvents showed protection against SGD-induced cell death while polar (H_2_O) and non-polar (*n-*hexane) fractions did not show significant effects. The protective effect of semi polar fraction (CH_2_Cl_2_, EtOAc, BuOH and MeOH) seems to be related to the presence of phytochemicals with semipolar nature. 

Different classes of pharmacologically active phytochemicals like alkaloid (violin) and a glycoside (viola quercitrin), flavonoids, caffeic acid derivatives, salicylic acid and triterpenoids have been isolated from the genus *Viola. *There are numerous works done on the chemicals present in various species of the genus *Viola. Viola odorata *is reported to contain alkaloids, steroids, tannins, phenolics, flavonoids glycoside, gaultherin, violutoside, flavonoids, aodoratine, coumarins, saponins, methyl salicylate, mucilage and vitamin C. The aerial parts and roots of *V. odorata *have about 30 cyclotides. Phenolic and flavonoid compounds were isolated from MeOH extract of the leaves of *V. odorata* (Hockenbery et al., 1993; Chandra et al., 2015)

In conclusion, the results of the present study showed that MeOH extract and semi polar fractions of* V. odorata *protect neuronal cells against SGD-induced cell death through antioxidant mechanisms and modification of Bax to Bcl-2 ratio.

## Conflict of interest

There is no conflict of interest about this article.
